# Chasmophyte associated stress tolerant bacteria confer drought resilience to chickpea through efficient nutrient mining and modulation of stress response

**DOI:** 10.1038/s41598-024-58695-3

**Published:** 2024-05-28

**Authors:** Sudipta Das, Hillol Chakdar, Adarsh Kumar, Rajni Singh, Anil Kumar Saxena

**Affiliations:** 1 ICAR-National Bureau of Agriculturally Important Microorganisms (NBAIM), Mau, Uttar Pradesh 275103 India; 2 Amity Institute of Microbial Technology, Amity University, Noida, Uttar Pradesh India

**Keywords:** Bacteria, Chasmophyte, Chickpea, Drought, Plant growth promotion, Soil microbiology, Applied microbiology, Environmental microbiology

## Abstract

In the present study, ten (10) selected bacteria isolated from chasmophytic wild *Chenopodium* were evaluated for alleviation of drought stress in chickpea. All the bacterial cultures were potential P, K and Zn solubilizer. About 50% of the bacteria could produce Indole-3-acetic acid (IAA) and 1-aminocyclopropane-1-carboxylate (ACC) deaminase. The bacteria showed wide range of tolerance towards pH, salinity, temperature and osmotic stress. *Bacillus paralicheniformis* L38, *Pseudomonas* sp. LN75, *Enterobacter hormachei* subsp. *xiangfengensis* LJ89, *B. paramycoides* L17 and *Micrococcus luteus* LA9 significantly improved growth and nutrient (N, P, K, Fe and Zn) content in chickpea under water stress during a green house experiment conducted following a completely randomized design (CRD). Application of *Microbacterium imperiale* LJ10, *B. stercoris* LN74, *Pseudomonas* sp. LN75, *B. paralicheniformis* L38 and *E. hormachei* subsp. *xiangfengensis* LJ89 reduced the antioxidant enzymes under water stress. During field experiments conducted following randomized block design (RBD), all the bacterial inoculations improved chickpea yield under water stress. Highest yield (1363 kg ha^−1^) was obtained in plants inoculated with *Pseudomonas* sp. LN75. *Pseudomonas* sp. LN75, *B. paralicheniformis* L38 and *E. hormachei* subsp. *xiangfengensis* LJ89 have potential as microbial stimulants to alleviate the water stress in chickpea. To the best of our knowledge this is the first report of using chasmophyte associated bacteria for alleviation of water stress in a crop plant.

## Introduction

Climate change can significantly affect the hydrological cycles, water balance and run-off characteristics in the arid and semi-arid regions. The increasing atmospheric temperature, erratic rain fall and rising sea levels under the changing climate scenario can significantly influence the availability of fresh waters required for agricultural production. Stress caused by scarcity of water (drought), salinity or high temperatures is outstanding environmental stress, which severely impairs crop production on irrigated land worldwide. More than 60% of the areas forming part of India’s arid and semi-arid ecosystems are affected by drought^1^.

Chickpea is one of the most important pulse crops of the Indian subcontinent (India, Pakistan, Bang Ladesh, Myanmar, and Nepal), contributing > 70% of the global production^2^. This important legume is mainly grown under rain-fed conditions and due to insufficient and erratic rainfall, water limitation becomes a major challenge during its life cycle. The intensity and duration of drought stress, the growthstage of the crop and genetic makeup determine the extent of yield loss due to drought. For example, drought stress during the reproductive phase resulted in a decrease in chickpea yield by 45–60%^3^. An increase in temperature by 0.1 °C and a decrease in rainfall by ~ 31% reduced the Chickpea yield by ~ 40 kg ha^−1^ in the Bundelkhand region^4^. Development of drought-tolerant varieties, shifting the sowing time and resource management practices are some of the strategies to manage drought stress in chickpea^5^. However, the majority of these alternatives are cost-intensive and are beyond the reach of resource-poorfarmers.

Microbes due to their versatile metabolic functions and ability to develop a beneficial association with plants can be very helpful to combat abiotic stress in plants. Many of the soil and plant-associated bacteria are known to produce several phytohormones like auxin, cytokinin, and gibberellins; enzymes like aminocyclopropane-1-carboxylic acid (ACC) deaminase which can directly help plants to cope with several abiotic stresses including drought^6^. Microorganisms can help in improving plant nutrition by solubilization and mineralization of unavailable nutrient sources which in turn can improve stress resilience^7^. Besides, several bacteria have been reported to modulate genes involved in the drought response of plants^8,9^. A variety of bacterial genera like *Bacillus*, *Pseudomonas*, *Azotobacter*, *Serratia*, *Achromobacter*, *Arthrobacter*, and *Microbacterium* have been reported to improve drought resilience of several crop plants^10^. Bacteria like *B. subtilis*, *B. megaterium*, *B. amyloliquefaciens*, *Pseudomonas putida* have also been reported to improve drought tolerance of chickpea. Even inoculation of bacteria like *B. mojavensis*, *Enterobacter cloacae* and *Providencia vermicola* isolated from desert soil could improve the mineral nutrient acquisition in Chickpea under water limited conditions^11^. He et al. also reported that *Pseudomonas* sp. and *Bacillus* sp*.* isolated from the rhizosphere of *Haloxylon ammodendron* growing in the Tengger desert of China also alleviated drought stress in ryegrass^12^. Thus, microorganisms associated with xerophytes or other extremophytes surviving under water-limited conditions have tremendous potential for stress alleviation of crop plants. However, the reports on using extremophyte-associated microbes for drought alleviation of crop plants are limited.

Chasmophytes are a type of extremophytes which grow in the cracks and crevices of rocks. Thus, the microorganisms associated with such plants might help them to adapt and survive under water and nutrient limited conditions of stone cracks. Such microorganisms can find applications to develop sustainable technologies to alleviate water stress in crop plants. To test this hypothesis in the present study, we characterized and evaluated ten (10) selected bacteria isolated earlier from chasmophytic wild Chenopodium plants collected from the Ladakh region of India for their potential to alleviate water stress in Chickpea.

## Results

### Characterization of bacteria for plant growth promoting traits

Ten selected bacterial cultures belonging to *Bacillus, Micrococcus, Enterobacter, Pseudomonas* and *Microbacterium* were subjected to screening for various plant growth promoting traits. All the bacterial cultures could solubilize at least two of the insoluble P sources (Table 1). The studied isolates could solubilize iron phosphate in the range of 2.28 to 24.08 mg L^−1^*.* On the other hand, solubilization of rock phosphate ranged from 0.56 to 25.19 mg L^−1^. *B. stercoris* LN74 showed highest solubilization of iron (24.08 ± 0.003 mg L^−1^) and rock phosphate (25.19 ± 0.006 mg L^−1^). In general, aluminium phosphate was solubilized to lesser extent (0.49 to 6.85 mg L^−1^) as compared to iron or rock phosphate by all the isolates. *Microbacterium* sp. LN19 recorded the maximum solubilization of aluminium phosphate (6.85 ± 0.059 mg L^−1^) (Table 1). All the isolates could solubilize potassium in a range of 57.6 to 74.1 ppm and the highest value was recorded for *B. paramycoides* L17 (74.10 ± 0.987 ppm) followed by *Pseudomonas* sp. LN75 (69.0 ± 0.897 ppm). Zinc solubilization by the bacterial cultures ranged between 3.8 to 25.6 ppm with highest being recorded for *B*. *paramycoides* L17 (25.6 ± 0.057 ppm) (Table 1). Production of IAA was exhibited only by five bacteria viz. *Micrococcus luteus* LA9, *B. stercoris* LN74, *Enterobacter hormachei* subsp. *xiangfengensis* LJ89, *Pseudomonas* sp. LN75, *M. imperiale* LJ10 with highest value recorded for *Pseudomonas* sp. LN75 (116.53 µg mL^−1^). Among the 10 isolates screened, only four isolates viz. *Micrococcus luteus* LA9, *Enterobacter hormachei* subsp. *xiangfengensis* LJ89, *Microbacterium* sp. LN19 and *M. imperiale* LJ10 were found to be positive for ACC deaminase (Table 1).

**Table 1:** Plant growth promoting attributes of the selected isolates.

Isolate	Solubilized P (mg L^−1^)			Solubilized K (ppm)	Solubilized Zn (ppm)	IAA production (µg mL^−1^)	ACC deaminase activity
	IP	AP	RP				
*Bacillus siamensis* L32	2.28 ± 0.005^e^	ND	1.25 ± 0.007^g^	67.7 ± 0.976^c^	4.6 ± 0.021^e^	ND	−
*Micrococcus luteus* LA9	2.39 ± 0.008^e^	ND	3.26 ± 0.015^e^	62.7 ± 0.876^d^	ND	12.73^d^	+
*B. paralicheniformis* L38	3.58 ± 0.008^d^	ND	2.29 ± 0.008^f^	62.6 ± 0.912^d^	9.87 ± 0.0487^d^	ND	−
*B. stercoris* LN74	24.08 ± 0.003^a^	0.49 ± 0.001^c^	25.19 ± 0.006^a^	58.0 ± 0.789^e^	3.8 ± 0.0147^f^	40.60^c^	−
*B. cereus* L34	2.47 ± 0.007^e^	4.90 ± 0.038^b^	4.40 ± 0.015^d^	57.6 ± 0.898^e^	24.6 ± 0.09^a^	ND	
*B. paramycoides* L17	8.18 ± 0.026^b^	ND	1.04 ± 0.002^ g^	74.1 ± 0.987^a^	25.6 ± 0.057^a^	ND	−
*Enterobacter hormachei* subsp. *xiangfengensis* LJ89	2.65 ± 0.006^e^	0.42 ± 0.004^c^	0.759 ± 0.006^h^	66.6 ± 0.976^c^	15.07 ± 0.048^c^	107.60^b^	+
*Pseudomonas* sp. LN75	4.84 ± 0.004^c^	0.50 ± 0.001^c^	0.56 ± 0.002^h^	69.0 ± 0.897^b^	3.8 ± 0.017^f^	116.53^a^	−
*Microbacterium* sp. LN19	5.57 ± 0.001^c^	6.85 ± 0.059^a^	5.72 ± 0.034^c^	66.2 ± 0.678^c^	21.07 ± 0.057^b^	ND	+
*M. imperiale* LJ10	7.94 ± 0.044^b^	0.37 ± 0.003^c^	13.19 ± 0.077^b^	58.9 ± 0.980^e^	21.93 ± 0.067^b^	15.67^d^	+

#Numerical values presented in the table are mean ± standard error.

*IP* iron phosphate, *AP* aluminium phosphate, *RP* rock phosphate, *ND* not detected.

Means with same letters are not significantly different at 95% confidence level.

### Environmental tolerance of the selected isolates

The tested bacterial isolates showed wide variation in environmental tolerance (Table 2). All the bacterial isolates could tolerate high pH (11.0) except *B. cereus* L34. All the isolates could tolerate at least up to 5% NaCl (w/v). *B. cereus* L34 and *B. paraparalicheniformis* L38 showed tolerance to 15% NaCl. All bacterial isolates except *Micrococcus luteus* LA9, *B. stercoris* LN74 and *Microbacterium* sp. LN19 could grow at 4 °C. The cultures when incubated at 50 °C, all except *Pseudomonas* sp. LN75, *Enterobacter hormachei* subsp. *xiangfengensis* LJ89, *B. cereus* L34 exhibited growth. All bacterial isolates could luxuriantly grow in presence of 30% PEG6000 (Table 2).

**Table 2:** Environmental tolerance of the selected isolates.

Isolate	pH tolerance	Salinity (%NaCl) tolerance	Temperature (°C) tolerance	Growth with 30% PEG 6000
*Bacillus siamensis* L32	7–11	5–10	4–50	+
*Micrococcus luteus* LA9	7–11	0–5	35–50	+
*B. paralicheniformis* L38	–	5–15	35–50	+
*B. stercoris* LN74	7–11	5–10	35–50	+
*B. cereus* L34	7–9	5–15	4–35	+
*B. paramycoides* L17	6–11	0–5	4–50	+
*Enterobacter hormachei* subsp. *xiangfengensis* LJ89	6–11	5–10	4–35	+
*Pseudomonas* sp. LN75	6–11	0–5	4–35	+
*Microbacterium* sp. LN19	6–11	0–5	35–50	+
*M. imperiale* LJ10	6–11	0–5	4–50	+

### Green house experiment to evaluate the potential of bacteria to alleviate water stress in chickpea

Water stress significantly affected the growth of chickpea plants (Table 3). However, inoculation of bacteria had positive effects on plant growth and could effectively alleviate the harmful effects of water stress. All the treatments with bacterial inoculation significantly improved the root length in comparison to the uninoculated stressed plants (at 30% FC). The root length of the chickpea plants under water stress was significantly improved by inoculation of *B. paralicheniformis* L38 (19.34 ± 0.410 cm) (Table 3). However, the bacterial inoculation treatments did not show any significant differences among themselves. Significant improvement in root architecture of chickpea plants under water stress (30% FC) was observed following the bacterial inoculation. Highest root surface area (36.11 ± 0.11 cm^2^) was observed in case of the plants inoculated with *Micrococcus luteus* LA9. In addition to the treatment with *M*. *luteus* LA9, plants inoculated with *B. paramycoides* L17 (21.65 ± 0.02 cm^2^), *Bacillus siamensis* L32 (23.84 ± 0.25 cm^2^) and *Pseudomonas* sp. LN75 (20.99 ± 0.47 cm^2^) showed significantly higher root surface area than the stressed (30% FC) as well as non-stressed (60% FC) uninoculated plants (Table 3). Formation of secondary roots in the inoculated plants under stressed conditions (30% C) was significantly higher than that of their uninoculated counterparts. Highest number of links (666.00 ± 26.27) and forks (265.67 ± 8.95) were observed in the plants inoculated with *Micrococcus luteus* LA9 followed by *B. siamensis* L32 (Table 3). Majority of the treatments were statistically on par with respect to root dry weight of the chickpea plants. The root dry weight of the plants inoculated with *B. paralicheniformis* L38 was significantly higher as compared to that of *B. cereus* L34. Under water stress (30% FC), a significant increase in the shoot length was observed in inoculated plants as compared to the un-inoculated ones (Table 3). Highest shoot length (23.56 ± 0.46 cm) was recorded with the inoculation of *B. paralicheniformis* L38. Similarly, bacterial inoculation significantly improved the shoot dry weight under water stress and inoculation of *B. siamensis* L32 resulted in the highest (0.99 ± 0.02 g) dry weight (Table 3).

**Table 3:** Effect of microbial inoculation on plant biometric parameters of chickpea (var. Pusa 362) grown under water stress.

Treatments	Root parameters					Shoot parameters	
	Length (cm)	Mean surface area (cm^2^)	Average no. of links	Average no. of forks	Dry weight (g)	Length (cm)	Dry weight (g)
L38 + 30% FC	19.34 ± 0.410^a^	17.76 ± 0.16^e^	323.00 ± 6.65^c^	138.33 ± 13.56^c,d,e^	0.21 ± 0.039^a^	23.56 ± 0.46^a^	0.95 ± 0.05^a,b^
LA9 + 30% FC	18.80 ± 0.151^a,b^	36.11 ± 0.11^a^	666.00 ± 26.27^a^	265.67 ± 8.95^a^	0.15 ± 0.009^a,b^	21.27 ± 1.99^a,b,c^	0.92 ± 0.02^a,b^
LJ89 + 30% FC	17.61 ± 0.831^a,b^	14.41 ± 0.11^g^	169.67 ± 2.90^e^	63.00 ± 1.15^g^	0.16 ± 0.015^a,b^	21.80 ± 1.66^a,b,c^	0.92 ± 0.01^a,b^
L17 + 30% FC	17.71 ± 1.006^a,b^	21.65 ± 0.02^c^	325.67 ± 11.83^c^	122.33 ± 4.33^d,e^	0.18 ± 0.014^a,b^	21.95 ± 0.18^a,b,c^	0.93 ± 0.03^a,b^
LN75 + 30% FC	18.35 ± 0.950^a,b^	20.99 ± 0.47^c^	270.64 ± 8.01^d^	89.67 ± 3.18^f^	0.17 ± 0.013^a,b^	23.03 ± 0.88^a,b^	0.94 ± 0.06^a,b^
LN74 + 30% FC	16.97 ± 1.248^a,b^	11.84 ± 0.59^h^	214.33 ± 3.18^d^	83.00 ± 0.58^f^	0.14 ± 0.010^a,b^	19.29 ± 1.81^c,d^	0.87 ± 0.01^b^
L34 + 30% FC	16.71 ± 1.237^a,b^	17.21 ± 0.15^e,f^	327.33 ± 1.45^c^	120.67 ± 8.95^e^	0.13 ± 0.017^b^	21.45 ± 0.71^a,b,c^	0.88 ± 0.02^b^
L32 + 30% FC	17.51 ± 0.574^a,b^	23.84 ± 0.25^b^	424.67 ± 2.90^b^	184.33 ± 3.76^b^	0.19 ± 0.014^a,b^	22.54 ± 0.72^a,b,c^	0.99 ± 0.02^a^
LJ10 + 30% FC	17.19 ± 0.634^a,b^	16.79 ± 0.05^f^	321.00 ± 2.08^c^	145.00 ± 1.73^c^	0.21 ± 0.021^a,b^	20.99 ± 1.03^a,b,c^	0.96 ± 0.02^a,b^
LN19 + 30% FC	17.13 ± 0.356^a,b^	16.47 ± 0.10^f^	290.31 ± 4.40^c^	139.33 ± 1.45^c,d^	0.18 ± 0.016^a,b^	19.75 ± 0.64^b,c^	0.95 ± 0.02^a,b^
Control (60% FC)	16.27 ± 0.172^b^	20.01 ± 0.58^d^	296.00 ± 1.52^c^	133.67 ± 1.45^c,d,e^	0.18 ± 0.026^a,b^	20.86 ± 1.03^a,b,c^	0.92 ± 0.02^a,b^
Control (30% FC)	11.88 ± 0.172^c^	5.38 ± 0.53^i^	99.00 ± 4.04^f^	40.33 ± 0.89^h^	0.15 ± 0.032^a,b^	16.11 ± 0.64^d^	0.74 ± 0.02^c^

The results were recorded after 45 days of sowing.

#Numerical values presented in the table are mean ± standard error.

Means with same letters are not significantly different at 95% confidence level.

**Figure 1:**
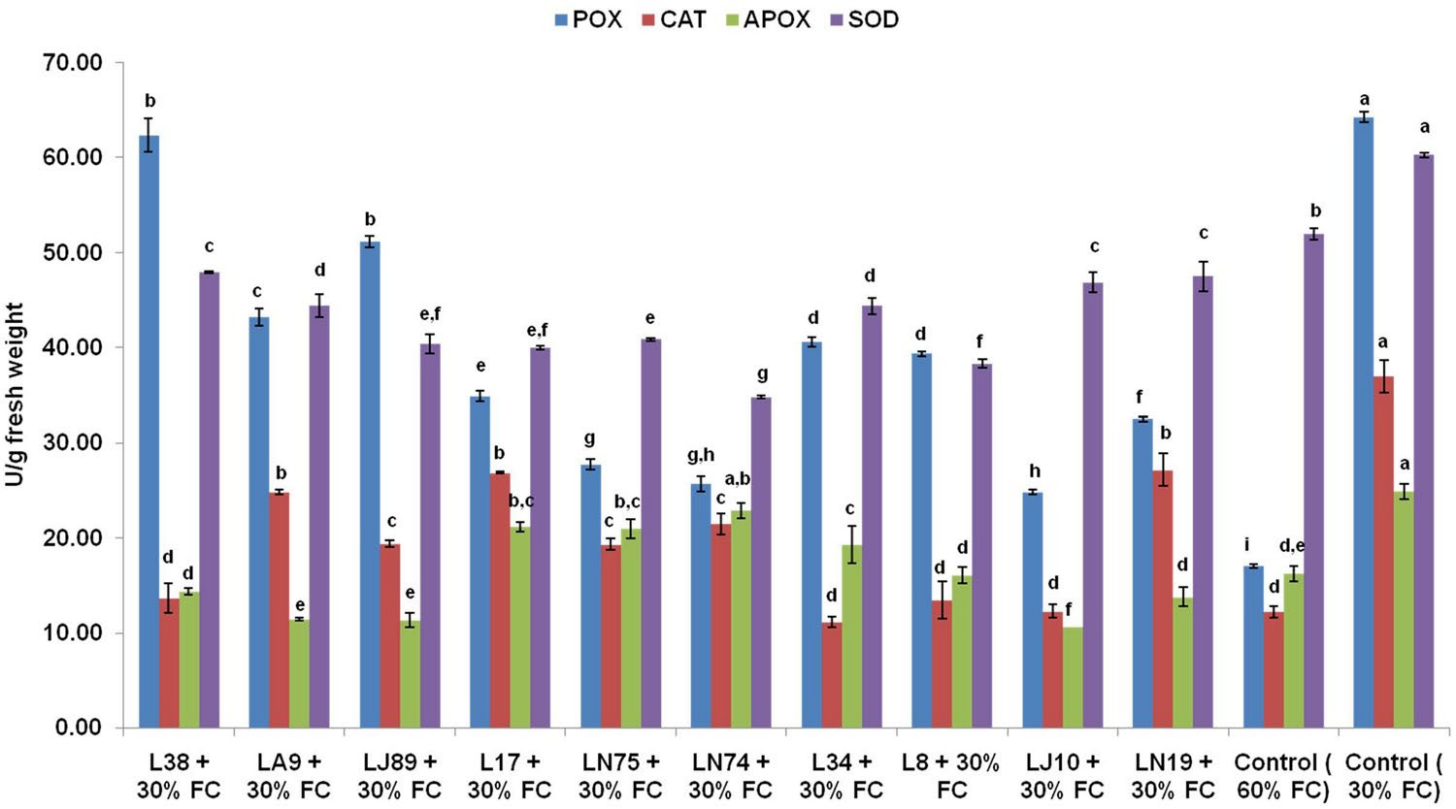
Effect of microbial inoculation on expression of various antioxidant enzymes in Chickpea leaves under water stress during greenhouse experiment. The error bars indicate standard error (SE) of mean. The letters placed above each bar indicate ranks obtained through Duncan’s multiple range test (DMRT); different letters represent significantly different mean values with 95% confidence. *FC* field capacity, *POX* peroxidase, *CAT* catalase, *APOX* ascorbate peroxidase, *SOD* superoxide dismutase.

Biochemical attributes of the chickpea plants growing under water stress were also improved significantly upon inoculation of bacteria (Table 4). Highest amount of chlorophyll *a*, *b* and carotenoids was recorded in the plants inoculated with *B. paralicheniformis* L38. Photosynthetic pigment content in the L38 inoculated plants was even better than that of non-stressed plants. Under water stress, the total protein content in the chickpea leaves also significantly increased upon bacterial inoculation under water stress. Except the treatments receiving inoculation with L38 and L32, all other treatments showed lower photosynthetic content as compared to the non-stressed (60% FC) plants (Table 4). Treatments with *B. siamensis* L32, *B. stercoris* LN74, *B. paralicheniformis* L38 and *M. imperiale* LJ10 and *Enterobacter hormachei* subsp. *xiangfengensis* LJ89 showed significantly higher amount of total protein even as compared to the stressed as well as non-stressed control plants (Table 4). The soluble sugar content in leaves was also significantly improved by bacterial inoculation under water stress. Highest amount of soluble sugar was recorded in the plants inoculated with *B. siamensis* L32 (92.77 ± 0.50 mg g^−1^ fresh weight) followed *Enterobacter hormachei* subsp. *xiangfengensis* LJ89 (92.05 ± 0.38 mg g^−1^ fresh weight), although the two treatments were statistically at par (Table 4). With regards to the proline accumulation, the highest value was recorded for un-inoculated plants grown under water stress (30% FC) (458.4 ± 10.40 µg g^−1^ fresh weight) and the proline content was much lower in the non-stressed plants (60% FC). Treatments inoculated with different bacterial cultures also showed significantly lower proline accumulation (Table 4). Lowest proline accumulation was observed in case of the plants inoculated with L38 and L34 which were however statistically at par. Antioxidant enzymes are important stress indicators. Highest POX, CAT, APOX and SOD activities were observed in the un-inoculated chickpea plants growing under 30%FC (Fig. 1). However, the antioxidant enzyme activities significantly decreased when the plants were grown with sufficient water (60% FC). Bacterial inoculation also reduced the activities of POX, CAT, APOX and SOD under water stress. Among the bacterial inoculation lowest (24.875 U g^−1^ fresh weight) peroxidase activity was recorded in plants inoculated with *M. imperiale* LJ10 followed by *B. stercoris* LN74 (25.113 U g^−1^ fresh weight) and *Pseudomonas* sp. LN75 (26.317 U g^−1^ fresh weight) (Fig. 1). Lowest (11.21 U g^−1^ fresh weight) catalase activity was recorded in *B. cereus* L34 which statistically at par with that of the non-stressed control plants (11.74 U g^−1^ fresh weight) (Fig. 1). Catalase activity in the stressed plants inoculated with L38, L8, LJ10 were statistically indifferent with the non-stressed plants (Fig. 1). In case of ascorbate peroxidise activity, *M. imperiale* LJ10 inoculated plants showed lowest (10.70 U g^−1^ fresh weight) activity (Fig. 1). Besides LJ10, inoculation with two other bacteria viz. *Micrococcus luteus* LA9 and *Enterobacter hormachei* subsp. *xiangfengensis* LJ89 showed low APOX activities which were significantly lower than that of the non-stressed control plants (10.91 U g^−1^ fresh weight). Lowest (34.84 U g^−1^ fresh weight) SOD activity was recorded in the plants inoculated with *B. stercoris* LN74 followed by *B. paramycoides* L17 (38.29 U g^−1^ fresh weight) and *E. hormachei* subsp. *xiangfengensis* LJ89 (39.12 U g^−1^ fresh weight) which were however, statistically at par with LN74 (Fig. 1). All the bacterial inoculation resulted in lower SOD activity even as compared to the non-stressed un-inoculated plants.

**Table 4:** Effect of microbial inoculation on pigment, protein and soluble sugar content of chickpea (var. Pusa 362) grown under water stress.

Treatments	*Chl a* (mg g^−1^)	*Chl b* (mg g^−1^)	Carotenoids (mg g^−1^)	Protein (mg g^−1^ f.w)	Soluble sugar (mg g^−1^ f.w)	Proline (µg g^−1^ f.w)
L38 + 30% FC	21.85 ± 0.07^a^	13.02 ± 0.31^a^	2.73 ± 0.04^a^	3.34 ± 0.05^a^	84.07 ± 0.29^c,d^	233.3 ± 7.63^g^
LA9 + 30% FC	16.99 ± 0.07^f^	8.07 ± 0.14^e^	1.92 ± 0.01^f^	2.94 ± 0.03^d^	88.89 ± 0.25^b^	282.4 ± 7.64^e,f^
LJ89 + 30% FC	13.63 ± 0.16^h^	7.53 ± 0.08^f,g^	1.74 ± 0.01^i^	3.08 ± 0.04^c^	92.05 ± 0.38^a^	371.8 ± 7.64^b^
L17 + 30% FC	17.59 ± 0.08^e^	9.74 ± 0.20^c^	1.98 ± 0.01^e^	2.96 ± 0.02^d^	84.58 ± 0.25^c^	273.7 ± 5.77^e,f^
LN75 + 30% FC	18.31 ± 0.04^d^	8.6 ± 0.04^d^	1.81 ± 0.02^h^	3.04 ± 0.02^c,d^	80.13 ± 0.76^e^	293.9 ± 7.63^d,e^
LN74 + 30% FC	15.2 ± 0.15^g^	7.28 ± 0.15^g^	1.86 ± 0.01^g^	3.28 ± 0.04^a^	85.01 ± 0.25^c^	293.9 ± 12.58^d,e^
L34 + 30% FC	20.48 ± 0.44^c^	9.57 ± 014^c^	2.07 ± 0.01^d^	3.05 ± 0.01^c,d^	80.99 ± 0.38^e^	233.3 ± 2.9^g^
L32 + 30% FC	21.18 ± 0.17^b^	11.3 ± 0.16^b^	2.21 ± 0.01^b^	3.34 ± 0.04^a^	92.77 ± 0.50^a^	262.2 ± 4.99^f^
LJ10 + 30% FC	10.7 ± 0.57^i^	7.66 ± 0.11^e,f^	1.93 ± 0.01^f^	3.17 ± 0.03^b^	78.69 ± 0.38^f^	354.5 ± 5.77^b,c^
LN19 + 30% FC	15.1 ± 0.08^g^	7.15 ± 0.12^g^	1.768 ± 0.03^i^	2.97 ± 0.01^d^	83.00 ± 0.62^d^	343.0 ± 2.88^c^
Control (60% FC)	21.3 ± 0.19^b^	9.3 ± 0.08^c^	2.12 ± 0.01^c^	2.74 ± 0.01^d^	72.94 ± 0.25^g^	305.5 ± 4.99^d^
Control (30% FC)	8.76 ± 0.06^j^	4.24 ± 0.02^h^	1.27 ± 0.01^j^	2.21 ± 0.03^e^	44.35 ± 0.52^h^	458.4 ± 10.40^a^

The results were recorded after 45 days of sowing.

#Numerical values presented in the table are mean ± standard error.

Means with same letters are not significantly different at 95% confidence level.

**Figure 2:**
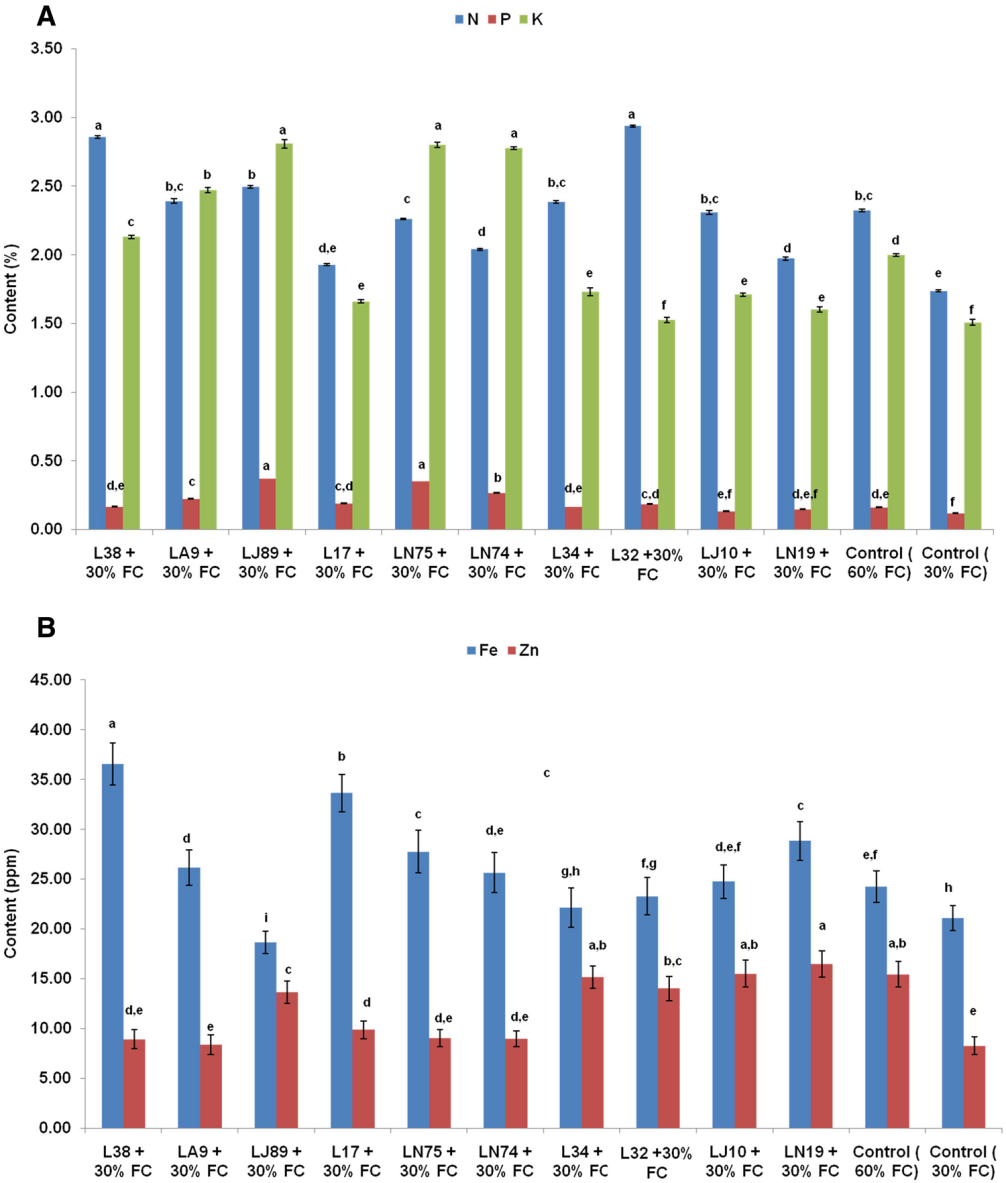
Effect of microbial inoculation on accumulation of (**A**) major (N, P, K) and (**B**) minor (Fe, Zn) nutrients in chickpea shoots under water stress during greenhouse experiment. The error bars indicate standard error (SE) of mean. The letters placed above each bar indicate ranks obtained through Duncan’s multiple range test (DMRT); different letters represent significantly different mean values with 95% confidence.

Stressed and non-stressed plants differed significantly in terms of nutrient contents. Analyses of macro and a few micronutrients of the inoculated plants grown under water stress showed significant improvement as compared to the un-inoculated ones (Fig. 2A,B). The nitrogen content was significantly higher in the plants inoculated with *B. siamensis* L32 (2.94%) or *B. paralicheniformis* L38 (2.86%) as compared to both stressed (1.74%) as well as non-stressed plants (2.32%) (Fig. 2A). Majority of the bacterial inoculations (except LJ10 and LN19) under water stress resulted in significantly higher P content as compared to their un-inoculated counterparts (Fig. 2A). Plants inoculated with *Enterobacter hormachei* subsp. *xiangfengensis* LJ89 recorded the highest P (0.37%) and K (2.81%) content followed by treatment inoculated with *Pseudomonas* sp. LN75 (P: 0.35% and K: 2.80%). Water stress significantly reduced the accumulation of Fe and Zn which was however, improved at variable degrees through inoculation of the bacteria. Highest Fe accumulation was observed in the plants inoculated with *B. paralicheniformis* L38 (36.58 ppm) followed by *B. paramycoides* L17 (33.65 ppm) and *Microbacterium* sp. LN19 (28.83 ppm) which were significantly higher than both stressed (21.09 ppm) as well as non-stressed plants (24.26 ppm) (Fig. 2B). On the other hand, maximum Zn accumulation was recorded in the plants inoculated with *Microbacterium* sp. LN19 (16.46 ppm) followed by the treatment inoculated with *M. imperiale* LJ10 (15.50 ppm) which were again significantly higher than stressed (8.25 ppm) and non-stressed (15.45 ppm) (Fig. 2B).

Based on the effects on overall plant growth, modulation of antioxidants, osmolytes and increased nutrient uptake in chickpea under greenhouse experiment, five bacteria viz. *B. paralicheniformis* L38, *Pseudomonas* sp. LN75, *E. hormachei* subsp. *xiangfengensis* LJ89, *B. paramycoides* L17 and *M*. *luteus* LA9 were selected for further evaluation under field conditions.

### Evaluation of selected isolates for alleviation of water stress in chickpea under field conditions

**Figure 3:**
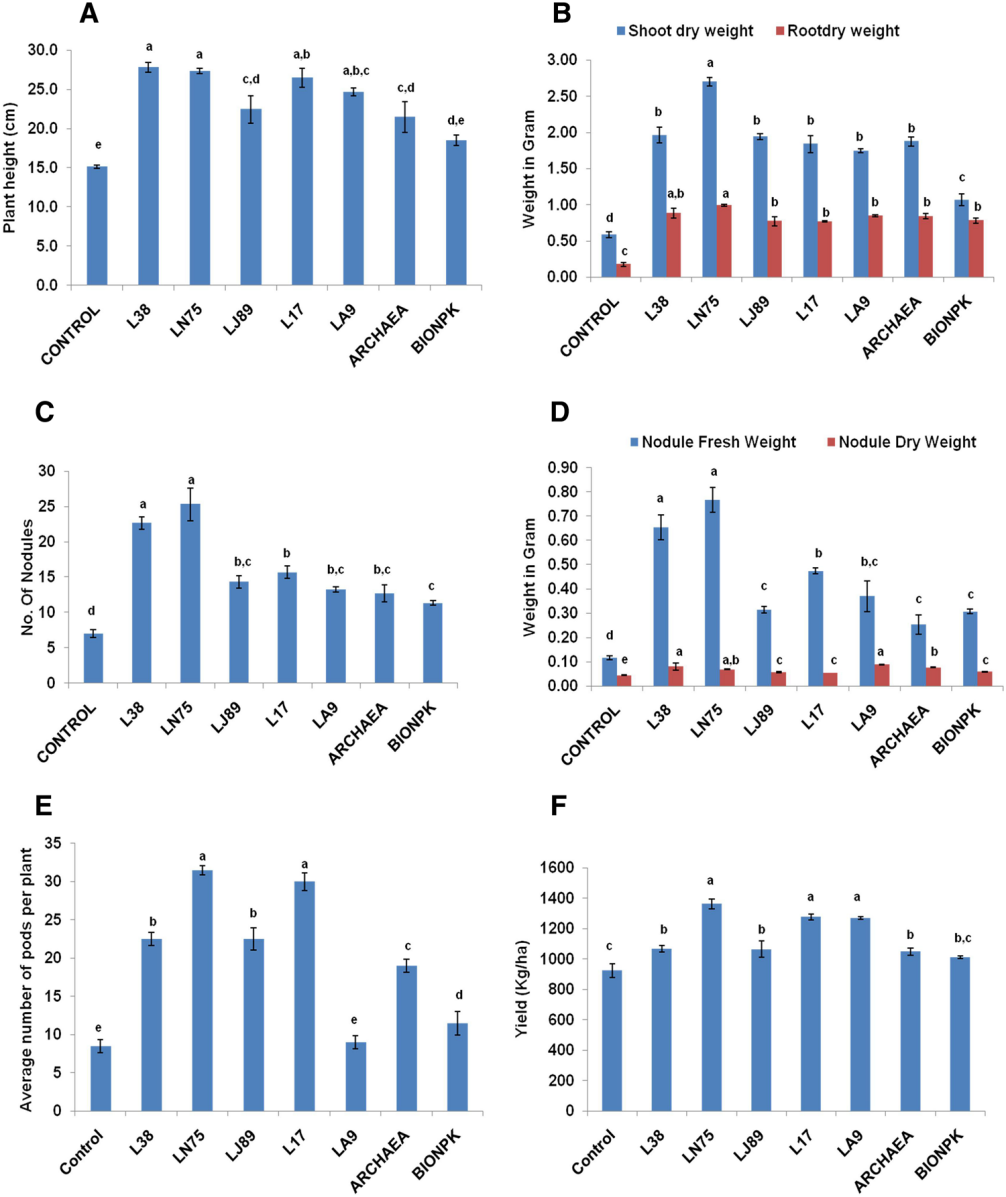
Effect of microbial inoculation on (**A**) plant height, (**B**) shoot and root dry weight, (**C**) number of nodules per plant, (**D**) nodule fresh and dry weight per plant, (**E**) average number of pods per plant, and (**F**) seed yield of chickpea under water stress during field evaluation. Plant height, root and shoot dry weight, number of nodules, nodule fresh and dry weight per plant were recorded at 45 days after sowing while yield parameters like number of pods per plant and seed yield were recorded during harvesting. The error bars indicate standard error (SE) of mean. The letters placed above each bar indicate ranks obtained through Duncan’s multiple range test (DMRT); different letters represent significantly different mean values with 95% confidence.

**Figure 4:**
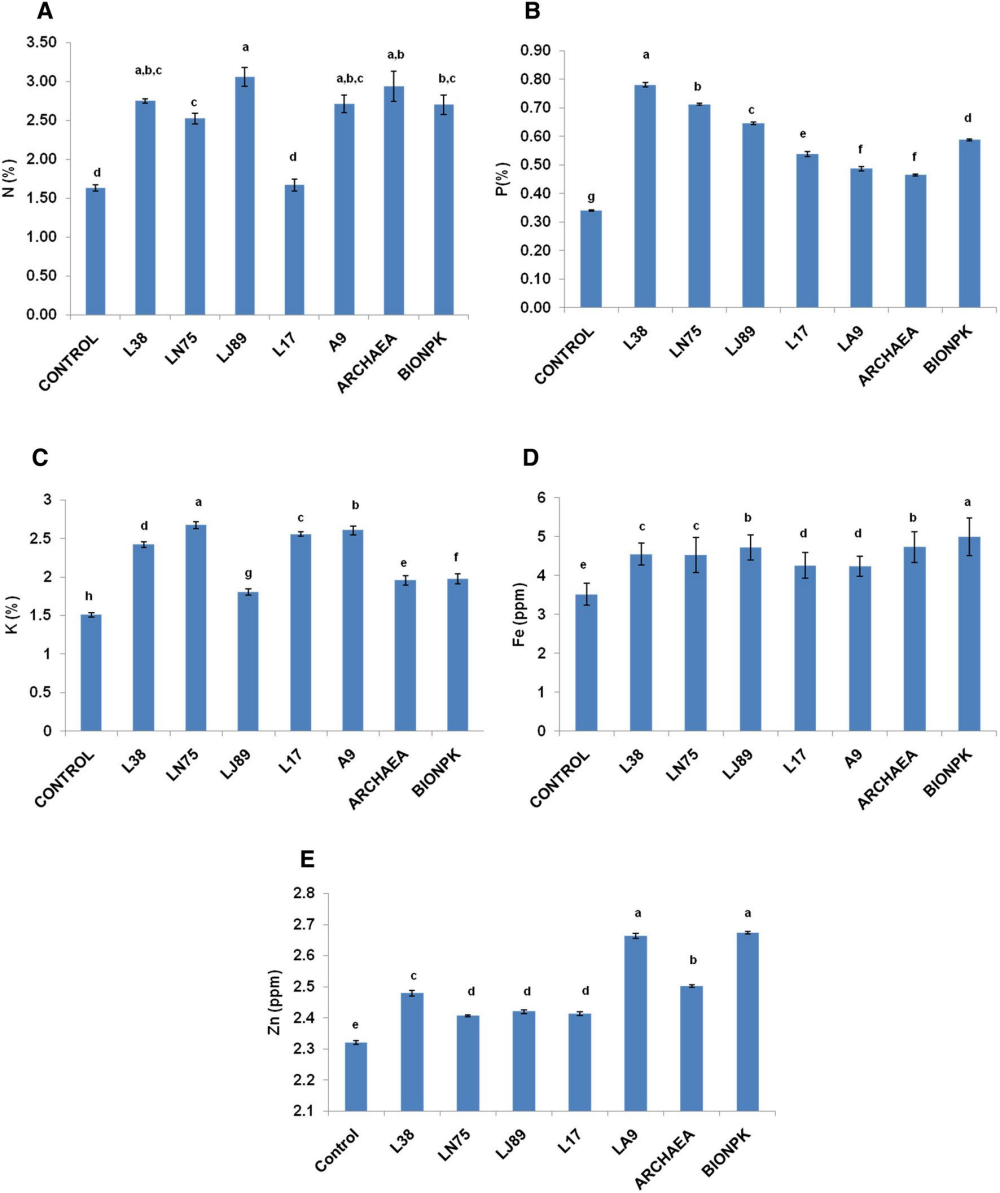
Effect of microbial inoculation on accumulation of (**A**) nitrogen (N), (**B**) phosphorus (P), (**C**) potassium (K), (**D**) iron (Fe), and (**E**) zinc (Zn) in the shoots of the chickpea at harvest stage. The error bars indicate standard error (SE) of mean. The letters placed above each bar indicate ranks obtained through Duncan’s multiple range test (DMRT); different letters represent significantly different mean values with 95% confidence.

During field evaluation, observations were recorded at 45 days after sowing (DAS) and at harvest. At 45 DAS, the plants inoculated with *B. paralicheniformis* L38 were recorded with the highest plant height (27.8 cm) which was significantly higher as compared the stressed control (15.1 cm). In plants treated with *B. paralicheniformis* L38, there was almost ~ twofold increase in the height in comparison to the control plants (Fig. 3A). The influence of L38 on plant height was however statistically on par with that of *Pseudomonas* sp. LN75 (Fig. 3A). The effect of inoculation with *Pseudomonas* sp. LN75 on root and shoot dry weight was significantly higher than that of the rest of the treatments (Fig. 3B). Inoculation of *Pseudomonas* sp. LN75 resulted in ~ fivefolds increase in the plant biomass (root and shoot dry weight) and nodule fresh weight as compared to the un-inoculated control (Fig. 3B,D). In comparison to the un-inoculated control, inoculation of *Pseudomonas* sp. LN75 increased the number of nodules, and nodule fresh weight by > 2.5 folds (Fig. 3C,D). At harvesting stage, highest number of pods (31.5 per plant) was recorded with the plants inoculated with *Pseudomonas* sp. LN75 followed by *B. paramycoides* L17 (Fig. 3E). All the bacterial inoculations led to significant improvement in the yield of chickpea under water stress (Fig. 3F). Highest yield (1363 kg ha^−1^) was obtained in case of the plants inoculated with *Pseudomonas* sp. LN75 followed by *B. paramycoides* L17 (1276 kg ha^−1^) and *Micrococcus luteus* LA9 (1067 kg ha^−1^) (Fig. 3F). However, these three treatments were statistically at par in terms of yield. The increment in yield due to inoculation of *Pseudomonas* sp. LN75 was ~ 1.47 and 1.29 times as compared to un-inoculated and archaea treated plants respectively. N, P, K, Fe and Zn contents in plant shoots and seeds also improved due to inoculation of bacteria under water stress (Fig. 4A–E). Highest N content was recorded in the shoots (3.06%) and seeds (2.40%) of the plants inoculated with *Enterobacter hormachei* subsp. *xiangfengensis* LJ89 (Fig. 4A, Table 5). Inoculation of *B. paralicheniformis* L38 resulted in highest shoot (0.78%) accumulation of P while highest P content in seeds (0.55%) was observed in plants inoculated with BioNPK (Fig. 4B). Accumulation of K was recorded highest (2.67%) in the shoots of the plants inoculated with *Pseudomonas* sp. LN75 followed by *Micrococcus luteus* LA9 (2.61%) (Fig. 4C). On the other hand, highest accumulation K (2.31%) in seeds was observed in plants inoculated with *Micrococcus luteus* LA9 which was however statistically indifferent from L17 and LN75 (Table 5). Highest shoot (4.99 ppm) and seed (2.92 ppm) accumulation of Fe was recorded in case of BioNPK inoculated plants (Fig. 4D, Table 5). Zn accumulation in shoots (2.67 ppm) and seeds (1.77 ppm) was also maximum in the plants inoculated with BioNPK (Fig. 4E, Table 5).

**Table 5:** Content of different macro and micronutrients in chickpea seeds.

Treatments	N (%)	P (%)	K (%)	Fe (ppm)	Zn (ppm)
L38	2.12 ± 0.04^c^	0.49 ± 0.017^b^	1.97 ± 0.06^b^	2.8 ± 0.07^b^	1.43 ± 0.04^c^
LA9	2.24 ± 0.03^b^	0.50 ± 0.013^b^	2.31 ± 0.04^a^	2.72 ± 0.05^c^	1.60 ± 0.05^b^
LJ89	2.40 ± 0.06^a^	0.50 ± 0.014^b^	1.31 ± 0.07^d^	2.77 ± 0.04^b^	1.42 ± 0.04^c^
L17	1.47 ± 0.03^d^	0.44 ± 0.011^c^	2.17 ± 0.05^a^	2.52 ± 0.04^d^	1.37 ± 0.05^d^
LN75	2.19 ± 0.05^b^	0.42 ± 0.012^c^	2.23 ± 0.03^a^	2.77 ± 0.05^b^	1.40 ± 0.04^c^
Control	1.25 ± 0.04^e^	0.22 ± 0.014^e^	1.41 ± 0.04^c^	2.67 ± 0.03^c^	1.22 ± 0.03^e^
Archaea	2.22 ± 0.06^b^	0.32 ± 0.015^d^	1.38 ± 0.04^c^	2.83 ± 0.05^b^	1.24 ± 0.03^e^
BioNPK	2.21 ± 0.05^b^	0.55 ± 0.017^a^	1.43 ± 0.03^c^	2.92 ± 0.04^a^	1.77 ± 0.04^a^

#Numerical values presented in the table are mean ± standard error.

Means with same letters are not significantly different at 95% confidence level.

## Discussion

Currently, climate change is the biggest challenge for sustainable agriculture. Increasing atmospheric temperature, evaporation and transpiration can seriously impact the water availability for agriculture. Erratic rainfall and subsequent drought spells may further aggravate the water scarcity. Such conditions adversely affect the production of rainfed crops like chickpea. Under Indian conditions, it is recommended to provide two irrigations for chickpea, ideally one at branching and another at pod formation stage^13^. Such irrigations can supplement the water requirement of the crop; however, limited ground water can be an issue at many places. Drought can reduce the germination, photosynthetic activity, translocation of assimilates and reproductive functions. Water stress during reproductive stage can reduce the chickpea yield by 50%^2^. Considerable efforts have been made to develop drought tolerant varieties which however, require a long time. Further, water management practices and irrigation systems have also been developed to grow more crops with limited water. However, many of these technologies are still beyond the reach of resource poor farmers.

Microorganisms have long been used in agriculture and are considered as low cost, eco-friendly alternatives for crop production and protection. Besides use as biofertilizers and biopesticides, microbes have attracted considerable attention to be exploited as natural agent to alleviate many abiotic stresses. Especially the microorganisms isolated from extreme habitats can find excellent applications for water stress alleviation in crop plants. It has been reported that halophilic archaea isolated from salt pans of Runn of Kutch, India helped in growing wheat under water stress^14^. Similarly, ACC deaminase producing *Pseudomonas* sp. from the roots of a desert plant *Alhagi sparsifolia* helped the plants to withstand water stress^15^. Multiple plant beneficial traits like phytohormone production, ACC deaminase activity, nutrient solubilisation, nitrogen fixation, exoploysaccharide production exhibited by microorganisms help the plants directly or indirectly to withstand a range of abiotic stress conditions. In the present study, ten bacterial cultures isolated from chasmophytic (growing in cracks and crevices of rocks) pig weed (*Chenopodium album*) collected from Ladakh region of India were characterized for different plant growth promoting attributes and evaluated for alleviation of water stress in chickpea. Bacterial population colonizing such plants growing under water and nutrient limited conditions have beneficial effects for water and nutrient acquisition. The bacterial isolates belonging to *Bacillus*, *Micrococcus*, *Enterobacter*, *Pseudomonas* and *Microbacterium* could solubilise at least two of the very recalcitrant insoluble P sources viz. rock phosphate, iron phosphate and aluminium phosphate. All the studied bacteria were good K solubilizers and all but *Micrococcus luteus* LA9 showed Zn solubilization. About 50% of the isolates also had the ability to produce IAA and ACC deaminase activities. These isolates also showed wide range of tolerance towards pH, salinity, temperature and osmotic stress. Bacteria associated with Chasmophytes or other extremophytes should have the ability to tolerate wide range of stress and exhibit multifarious growth promoting attributes to support the survival of the plants. Zia et al. reported that *Cronobacter sakazakii* RF-4, *Pseudomonas balearica* RF-2 and *Proteus mirabilis* R2 isolated from native weeds and soils of Cholistan desert of Pakistan showed tolerance towards salt (~ 6–10% NaCl), PEG 6000 (20%) and also exhibited P/Zn/K solubilization, calcite degradation, IAA production and ACC deaminase production^16^. These multi-stress tolerant bacteria possessing plant beneficial attributes were effective to alleviate drought stress in wheat through modulating physiological system of plants and improving resilience through better nutrient uptake. Another study carried out in the Cholistan desert of Pakistan also reported isolation of exopolysaccharide, ACC deaminase and IAA producing; and P solubilizing *Providencia vermicola* RS-15, *Bacillus subtilis* RP-01, *Mesorhizobium ciceri* RZ-11, *B. mojavensis* RS-14, *Enterobacter cloacae* RP-08 from chickpea plants^11^. These bacterial isolates were able to improve chickpea growth and yield under different moisture regimes. In the present study also, *B. paralicheniformis* L38, *Pseudomonas* sp. LN75, *Enterobacter hormachei* subsp. *xiangfengensis* LJ89, *B. paramycoides* L17 and *Micrococcus luteus* LA9 significantly improved growth of chickpea under water stress (30% FC). Improved root length, surface area and lateral rootsas a result of the inoculation of these isolates might be due to exogenous supply of phytohormones by the bacteria in the root zone. Robust root architecture is very critical for tolerance to drought stress. During the field experiments also the inoculation of *B. paralicheniformis* L38 and *Pseudomonas* sp. LN75 led to significant increase (two to five folds) in plant height and biomass during vegetative stage (45DAS). According to Khan et al., inoculation of *Paenibacillus lentimorbus* B-30488 along with salicylic acid and calcium alginate improved the height and dry weight of chickpea by 29.64 and 20.18% respectively under drought simulated conditions in field^17^. Inoculation of osmotolerant endophytic *Cronobacter dublinensis* strain MKS-1 and *Shewanella putrefaciens* strain MCL-1showed increased root volume, length and surface area of Pearlmillet under drought stress through increasing the IAA content^18^. In the present study, increase in plant biomass, protein and soluble sugar content was possibly due to better water and nutrient uptake through improved root architecture and increased nutrient availability by microbial functions in rhizosphere. Further, increased content of photosynthetic pigments viz. chlorophyll and carotenoids improved photosynthetic activities leading to higher plant biomass. It has been reported that *Providencia* sp. TCR05 and *Proteus mirabilis* TCR20 improved biomass of maize under drought stress through improvement of plant photosynthetic efficiency by increasing chlorophyll content^19^. Inoculation of *Bacillus amyloliquefaciens* SQR9 have been reported to boost seedling development and photosynthetic pigments thereby improving plant resilience to salt stress^20^. In the present study, *B. paralicheniformis* L38 inoculation to chickpea led to 3 folds and twofolds increase in contents of chlorophyll and carotenoids. Interesting Ly, highest plant biomass of chickpea during greenhouse experiment was also obtained with inoculation of *B. paralicheniformis* L38 under 30% field capacity. Further, Zhang et al. reported that inoculation of *B. pumilus* improved the integrity of chloroplast and mitochondria in cells of *Glycyrrhiza uralensis* under drought conditions resulting in increased chlorophyll content and photosynthetic activities^21^. Cohen et al. reported improved plant biomass, proline levels and photosynthetic pigments in Arabidopsis thaliana inoculated with *Azospirillum brasilense*^22^. Improved nutrient acquisition under water stress is another factor which determines stress resilience. Nitrogen and phosphorus are vital for photosynthetic pigment biosynthesis and other major metabolic functions while potassium is crucial for stomatal regulation and translocation of photosynthetic assimilates. Especially, potassium is very crucial for drought tolerance in plants through its contribution in cell elongation, membrane stability, and activation of aquaporins, water uptake, stomatal regulation and osmotic adjustment^23^. Zhnag et al. reported that arbuscular mycorrhiza fungi (AMF) *Rhizopus irregularis* improved potassium uptake of *Lycium barbarum* in root and translocation from root to shoot under drought stress^24^. Application of AMF along with *Azotobacter* and *Azospirillum* enhanced the N and K content of *Valeriana officinalis* under water stress^25^. Inoculation of AMF or PGPR (*Pseudomonas fluorescens* and *P. putida*) in common myrtle resulted in significant increase in root N (151–138%), P (176–181%), K (114–112%) as compared to non-inoculated seedlings under severe drought stress (30% FC)^26^. In the present study, *B. siamensis* L32, *B. paralicheniformis* L38, *Pseudomonas* sp. LN75 and *Enterobacter hormachei* subsp. *xiangfengensis* LJ89 significantly improved the N, P and K content in chickpea plants under water stress. All these bacteria are prominent P and K solubilizer which have contributed to make more nutrients available in the root zone of chickpea. In the present study, inoculation of *B. paralicheniformis* L38, *Pseudomonas* sp. LN75 and *Enterobacter hormachei* subsp. *xiangfengensis* LJ89 again improved the N, P and K content in shoots of chickpea grown under water stress during the field experiment. In case of chickpea, nodulation by rhizobia can significantly influence the nitrogen nutrition through fixation of atmospheric nitrogen. However, drought adversely affects nodulation by native rhizobia. In the present work, the number of nodules, nodule fresh and dry weight increased in the chickpea plants inoculated with *B. paralicheniformis* L38 and *Pseudomonas* sp. LN75 under water stress during the field trial. To some extent, the improvement in the nitrogen content in the chickpea plants inoculated with these two bacteria can be due to better rhizobial infection by the native rhizobia. Improved root architecture may also contribute towards higher rhizobial infection^27^. Yadav and Verma also reported that inoculation of *Pseudomonas aeruginosa* along with *Rhizobium leguminosarum* to chickpea led to higher nodule number, biomass and concomitant increase in nitrogen content in grain and straw^28^. Sing Le or dual inoculation of diazotrophic *Bacillus subtilis* OSU-142 and phosphate solubilizing *B. megaterium*M-3 to chickpea was also reported to significantly induce the nodule formation in chickpea^29^. Higher uptake and accumulation N, P, K, Ca, B, Zn and Mn has been reported in the drought tolerant chickpea varieties indicating a correlation between tolerance and levels of these nutrients^30^. In the present study, the tested bacteria were able to improve the accumulation of micronutrients like Fe and Zn in the chickpea plants under water stress both during the green house and field trials. It has been reported that sufficient availability of Zn in plant cells can improve water relations, membrane stability, osmolyte accumulation, stomatal regulation and photosynthesis under water stress^31^. Further, improved Fe availability might have increased the chlorophyll content and thereby led to higher photosynthetic activities under water stress.

Under water deficit conditions, plants synthesize a number of compounds including sugars, sugar alcohols and amino acids known as compatible solutes or organic acids to maintain the osmotic potential of the cells. Under drought stress, osmolyte concentrations increase in the cells and are thus considered as an important stress indicator. The increase in concentration of proline in plants exposed to moisture stress helps the plant to overcome to overcome negative consequences of drought^32^. Inoculation of microorganisms can reduce the water stress in plants by improving the uptake of water, nutrients and modulating the physiological functions mainly through hormonal and gene regulation. Thus the plants inoculated with microorganisms showed lower osmolyte concentrations. Vishnupradeep et al. reported significant reduction in proline content in leaves of maize inoculated with *Providencia* sp. TCR05 and *Proteus mirabilis* TCR20 under drought stress as compared to the un-inoculated ones^19^. Similarly, reduced proline levels have also been reported in chickpea inoculated with *B. subtilis, B. thuringiensis* and *B. megaterium* along with salicylic and putrescine under drought stress^33^. The reduced proline level in the plants receiving microbial inoculation and plant growth regulator was attributed towards osmotic adjustments and improved cellular bioenergetics due to treatment. In the present study, inoculation of *B. cereus* L34 and *B. paralicheniformis* L38 resulted in ~ 50% reduction in proline contents in chickpea leaves under water stress as compared to the un-inoculated plants grown under same conditions. In addition to the higher expression of osmolytes under water stress, plants also up-regulate the anitoxidative enzymes to reduce the concentrations of reactive oxygen species formed in the plant cells under stress. Antioxidant enzymes are rather general combat mechanisms of plant cells deployed in most of the stress conditions and are also considered as other important stress indicators. However, microbial inoculation can both up- and down-regulate the antioxidant enzyme production under stress. Certain microbes can improve the production of scavenging enzymes to remove the ROS more efficiently from the cells while others can reduce the level of perceived stress through hormonal regulations and other modulation of physiological functions. In the present study, inoculation of *Microbacterium imperiale* LJ10, *B. stercoris* LN74, *Pseudomonas* sp. LN75, *B. paralicheniformis* L38 and *Enterobacter hormachei* subsp. *xiangfengensis* LJ89 significantly reduced the antioxidant enzymes like POX, CAT, APOX ad SOD. Significant increase in the activity of antioxidant enzymes due to inoculation of drought tolerant bacteria has been reported^33–35^. Khan et al. reported that theinoculation of *B. subtilis, B. thuringiensis* and *B. megaterium* along with salicylic and putrescine under drought stress significantly reduced the APOX (70% and 68%) and SOD (61% and 65%) activities in both drought tolerant and sensitive varieties of chickpea^33^. Inoculation of *Bacillus* spp. also resulted in significantly reduced APOX and glutathione reductase activities in maize under drought stress^36^.

In chickpea, water stress severely impairs the key physiological and biochemical processes including photosynthesis, transpiration, nutrient uptake which ultimately leads to reduced growth and yield. Drought stress can reduce the chickpea yield by up to 50%. Especially, water stress at reproductive stage can result in heavy yield loss. Number of pods and number of seeds per plant in chickpea are highly influenced by environmental stress which ultimately adversely affects the seed yield^37^. Therefore, managing the drought stress can significantly improve the chickpea yield. In the present study, microbial inoculations significantly improved number pods per plant and seed yield. Earlier, Aulakh et al. reported that combined inoculation of *Mesorhizobium ciceri* RZ-11, *Bacillus subtilis* RP-01 and *B. mojavensis* RS-14 resulted in 45–50% improvement in chickpea yield under drought stress^11^. The improvement in yield parameters was attributed towards the plant growth promoting traits of the bacterial inoculants like IAA production, ACC deaminase production, P solubilization. Similarly, Laranjeira et al. also reported significant improvement in yield of chickpea grown at 50% water requirement and inoculated with a combination of arbuscular mycorrhiza and plant growth promoting rhizobacteria which contributed through improved nutrient availability and phtyohormone production^38^. The bacterial isolates used in this study showed potential for P, K and Zn solubilization along with IAA and ACC deaminase production. Increased nutrient availability, robust root architecture induced by the inoculation of the bacterial cultures might have contributed in the yield improvement of chickpea under water stress.

Chasmophyte associated bacteria exhibit tolerance to different environmental stresses and possess multiple growth promoting traits. Inoculation of these isolates to crop plants like chickpea could alleviate the moisture stress and improve growth and yield of the plant. Bacterial isolates, in particular *Pseudomonas* sp. LN75, *B. paralicheniformis* L38 and *Enterobacter hormachei* subsp. *xiangfengensis* LJ89 could be developed as microbe-based technology to alleviate the water stress in chickpea. Multifarious plant growth promoting traits of these bacteria helped the plants to mine water and nutrients more efficiently leading to reduced effect of water stress on plants which were evident from the decreased accumulation of stress indicators like osmolytes and antioxidants. Therefore, the above said bacterial inoculants can be used for sustainably improving the chickpea yield under water limited conditions. To the best of our knowledge this is the first report of using chasmophyte associated bacteria for alleviation of water stress in a crop plant.

## Experimental

### Bacterial cultures and their growth

Ten (10) bacterial cultures previously isolated from chasmophytic wild Chenopodium from the Ladakh region (32° 54′ 40.39″ N and 78° 18′ 57.24″ E; Elevation: 4507 m from sea level) were used in this study (Table 6). These bacterial cultures showed significant improvement in chickpea growth and development during an in vitro study in our laboratory^39^. All bacterial cultures were revived on nutrient agar (HiMedia, India) and incubated at 30 ± 2 °C for 24–48 h. The revived cultures were grown in nutrient broth (HiMedia, India) and incubated under the aforementioned conditions for further use.

**Table 6:** Details of the bacterial cultures used in this study.

Name of the bacteria	Isolated from	NAIMCC accession number	NCBI GenBank accession
*M. imperiale* LJ10	Wild pigweed (*Chenopodium album*) plants growing on the mountains of Tsomoriri, Ladakh, India	NAIMCC-B-03019	MZ443901
*Micrococcus luteus* LA9		NAIMCC-B-03021	MZ443872
*B. paralicheniformis* L38		NAIMCC-B-03015	MZ424732
*Bacillus siamensis* L32		TB-3729	MZ424745
*B. cereus* L34		NAIMCC-B-03014	MZ417511
*B. paramycoides* L17		NAIMCC-B-03016	MZ416925
*Enterobacter hormachei* subsp. *xiangfengensis* LJ89		NAIMCC-B-03018	MZ416789
*Pseudomonas* sp. LN75		NAIMCC-B-03022	OL629458
*Microbacterium* sp. LN19		NAIMCC-B-03020	MZ443871
*B. stercoris* LN74		NAIMCC-B-03017	MZ416788

### Characterization of bacteria for plant growth promoting traits

#### Quantitative estimation of P solubilization

To quantify P solubilization, bacterial cultures were raised in modified Pikovskaya’s (PKV) broth containing yeast extract-0.50 g L^−1^; dextrose-10.0 g L^−1^, (NH_4_)_2_SO_4_-0.50 g L^−1^, KCl-0.20 g L^−1^, MgSO_4_⋅7H_2_O-0.10 g L^−1^, MnSO_4_, xH_2_O-0.0001 g L^−1^, with either of the three insoluble P sources viz. aluminum phosphate (AP), iron phosphate (IP), rock phosphate (RP) at a concentration of 5.0 g L^−1^^40^. Each of the freshly grown ten bacterial cultures (2.5%, v/v) was inoculated to 50 mL PKV broth containing the three insoluble P sources separately. The conical flasks containing the inoculated broth were incubated in a shaking incubator (I200R, Humanlab Instruments Co., Korea) for 5 days at 30 ± 2 °C under continuous agitation (120 rpm). After 5 days of incubation, 5 ml broth from each flask was centrifuged (R12C-PLUS, REMI, India) at 10,000 rpm for 5 min and the supernatant was collected to estimate P content following the method described by Fiske and Subbarow^41^. A control was set up without any inoculation to negate soluble phosphates in the medium. These experiments were carried out in triplicates and repeated three times independently.

#### Quantitative estimation of K solubilization

For the quantification of K solubilization by the bacterial cultures, Aleksandrov broth (HiMedia, India) containing potassium alumino silicate as a source of insoluble K was used. Freshly grown bacterial cultures (2.5%, v/v) were inoculated to 50 mL broth followed by an incubation of 5 days at 30 ± 2 °C under continuous agitation (120 rpm). After incubation, the culture suspension was centrifuged as described in the previous section and the supernatant was collected. Potassium content (measuring light emission at 766 nm) in the supernatant was determined by a flame photometer^42^. A control was set up without any inoculation to negate soluble potassium in the medium.

A standard curve prepared using KCl was used to calculate the concentration of K in the supernatants.

#### Quantitative estimation of Zn solubilization

Zn solubilization by the bacterial cultures was estimated by growing the cultures in modified PKV broth containing zinc oxide (ZnO, 1 g L^−1^)^43^. Each bacterium was inoculated separately to 50 mL of Modified Pikovskaya’s broth containing ZnO. After inoculation, the flasks were incubated for 5 days at 30 ± 2 °C under continuous agitation (120 rpm). Un-inoculated medium served as control for the analyses. After incubation, the bacterial suspension was centrifuged (10,000 rpm for 5 min at 4 °C) to obtain the supernatant for quantification of solubilized Zn. The crude supernatant was diluted 20 times with double distilled water and the concentration of Zn was determined using an atomic absorption spectrophotometer (AA-6880, Shimadzu, Japan)^43^.

#### Quantitative estimation of IAA (indole-acetic acid) production

All the bacterial cultures were inoculated separately to 50 mL tryptone broth in 100 mL conical flasks and incubated for 48 h as per the conditions mentioned in the previous sections. After incubation, the bacterial suspension (50 mL) was centrifuged as described in the previous section and the supernatant was collected to quantify IAA following the procedures described earlier by Bric et al.^44^.

#### Qualitative estimation of ACC deaminase activity

DF Salt minimal medium with ACC (procured from TCI, India) as the sole nitrogen source was used for detecting ACC deaminase activity in the bacterial cultures^45^. The methodology described earlier by Das et al. was used to detect the ACC deaminase production in the bacterial cultures^39^.

### Characterization of bacteria for environmental tolerance

#### Salinity tolerance

The ability of the bacteria to tolerate various salinity levels (5, 10 and 15% NaCl, w/v) was determined by inoculating 100 µL bacterial culture on nutrient agar (Hi Media) plates supplemented with 0–15% (w/v) NaCl^46^. Freshly grown bacterial cultures were spotted on the plates and incubated at 30 ± 2 °C for 48–72 h.

#### pH tolerance

The bacterial cultures were evaluated for their ability to grow under a varying range of pH (6–11) on nutrient agar plates^46^. Nutrient Agar (HiMedia) was prepared and the pH was adjusted from 6 to 11 (at a pH interval of 1) using 1 M NaOH. After solidification of NA plates, bacterial isolates were spot inoculated (100 µL) and incubated at 30 ± 2 °C for 48–72 h.

#### Temperature tolerance

The bacterial cultures were streaked on nutrient agar (HiMedia, India) plates and incubated at different temperatures viz. 4, 35 and 50 °C for 48–72 h^46^.

#### Osmotic tolerance

Nutrient broth supplemented with 30% Polyethylene g Lycol (PEG) 6000 (SRL, India) was used to screen the osmotic tolerance of the bacterial isolates. Each test bacteria was inoculated (2%, v/v) to one tube containing the medium and incubated on a shaking incubator at 120 rpm and 30 ± 2 °C for 5 days. The bacterial growth was determined by reading the absorbance at 600 nm against un-inoculated medium as a reference.

### Green house experiment for evaluating the bacteria for alleviation of water stress in chickpea

#### Pot experiment

A pot experiment under green house conditions was taken to evaluate the effect of the bacterial isolates on growth of chickpea under water limited conditions. Potting mixture was prepared by mixing sand and field soil in 1:3 ratio followed by sterilization through tyndallization. Plastic pots (12.0 cm) wiped with 70% ethanol were filled with 5.0 kg sterilized sand:soil mixture. The soil was collected from the experimental fields of ICAR-NBAIM, Mau. The physico-chemical characteristic and nutrient profile of the soil are as—pH: 8.3 ± 0.20, electrical conductivity: 0.85 ds m^−1^, organic carbon: 0.43%, N: 170 kg ha^−1^, P: 44.7 kg ha^−1^, K: 138.23 kg ha^−1^. Recommended dose of N (15 kg ha^−1^) and P (20 kg ha^−1^) was supplied with the potting mixture by adding 6.7 mg N kg^−1^ and 8.9 mg P kg^−1^ soil in the form of urea and sing Le superphosphate (SSP) to the soil. The pots were then saturated with water and the weights of the pots were recorded both before and after adding water to calculate the field capacity (FC)^47^. According Ly, 60% and 30% FC were calculated as applicable for different treatments (Table 3). Seeds of chickpea var. PUSA362 were surface sterilized using 2% NaOCl (HiMedia, India) followed by washing with ethanol (70%) for 3 min and finally washed thrice with sterile distilled water. Freshly grown log phase bacterial cultures (containing10^8^ CFU mL^−1^) were mixed with sterile 1.5% Carboxymethyl cellulose (CMC) as suggested by Singh et al. and used for coating of chickpea seeds^47^. Another set of seeds was treated with mixture of uninoculated nutrient broth and CMC and used as uninoculated control. Four seeds were sown in each pot at a depth of 1–1.5 cm. The pots were maintained at 26 ± 2 °C temperature, 60–70% relative humidity, and 12 h light/dark. The experiment was continued till 45 days after sowing (DAS). The water content in each pot was maintained as per the field capacity of the respective treatments. The experiment was conducted following a completely randomized design (CRD) with three replications of each treatment.

#### Determination of plant biometric parameters

After 45 DAS, the plants were harvested to record various biometric parameters like root length, shoot length, root and shoot dry weight. The root architecture was scanned using an EPSON LA2400 (3rd Gen.) scanner and parameters like surface area, number of links and forks were analyzed using WINRHIZO (V. 2017a) Program (Regent Instruments Inc. Ltd., Canada).

#### Estimation of chlorophyll, carotenoids, protein, soluble sugar and proline

Chlorophyll a (*Chl*a), chlorophyll b (*Chl*b), carotenoids, protein and soluble sugar content was determined using the fresh leaves of chickpea. For chlorophyll and carotenoid estimation, leaf samples (500 mg) were crushed in 80% acetone (Fisher Scientific, India) and filtered thoroughly with Whatman no. 1 filter paper (GE Healthcare, India). The filtrate was collected in 50 mL falcons tube (Tarsons, India) and volume was made up to 50 mL with 80% acetone. The tubes were were incubated at 65 °C for 4 h and after incubation the absorbance was recorded at 470,645,663 nm against 80% acetone blank. *Chl*a, *Chl*b and total carotenoids were calculated as per the equations given below^48^:

$${\text{Chl-a}} = {12}.{\text{7 A}}_{{{663}}} {-}{ 2}.{\text{69 A}}_{{{645}}} ,$$

$${\text{Chl-b}} = {22}.{\text{9A}}_{{{645}}} - {4}.{\text{68A}}_{{{663}}} ,$$

$${\text{Total carotenoids }} = \left[ {{1}000{\text{A}}_{{{47}0}} - \left( {{3}.{\text{27 chl-a}} + {1}0{\text{4 chl-b}}} \right)} \right]/{229}.$$

Protein content was determined following the methods described earlier by Lowry and soluble sugars were determined using the methods given by Dubois et al.^49,50^. To estimate the proline content, 0.5 g leaf samples were ground in 10 mL of 3% aqueous sulphosalicylic acid (HiMedia, India) and filtered through Whatman filer paper no. 1 (GE Healthcare, India). The filtrate was used to determine the proline content following the methods given by Ref.^51^.

#### Estimation of antioxidant enzymes

To extract antioxidant enzymes, 0.5 g of fresh leaves were ground in 5 mL phosphate buffer (50 mM) by placing it on ice bath. The extract was then centrifuged at 13,000×*g* for 20 min at 4 °C. The supernatant was collected and used for the determination of superoxide dismutase (SOD), catalase (CAT), peroxidase (POX) and ascorbate peroxidase (APOX) activities. SOD activity was determined by recording absorbance at 560 nm following the method described by Dhindsa et al.^52^. POX activities were measured following the protocols described earlier by Castillo et al.^53^. APOX and CAT activities were determined as described earlier by Nakano and Asada and Aebi respectively^54,55^.

### Nutrient analyses in shoots

N, P, K, Fe and Zn content in the shoot of the plants were determined. The shoot samples (0.5 g) were oven dried, ground and digested using a mixture of H_2_SO_4_ and 30% hydrogen peroxide (H_2_O_2_) in digestion tubes. The digests were filtered and subjected to nutrient analyses. N (%) was determined following the Kjeldahl method^56^. P(%) content was determined following the methods described by Olsen et al. while K(%) content in the digest was determined by flame photometric method^42,57^. The digests were diluted appropriately with double distilled water and subjected to determination of Fe and Zn using atomic absorption spectrophotometer (AA-6880, Shimadzu, Japan).

### Evaluation of selected isolates for alleviation of water stress in chickpea under field conditions

On the basis of pot experiment, five best performing strains (*B. paralicheniformis* L38, *Pseudomonas* sp. LN75, *E. hormachei* subsp. *xiangfengensis* LJ89, *B. paramycoides* L17 and *M*. *luteus* LA9) were evaluated under field conditions during 2021–2022. Two bacterial formulations viz. BioNPK comprised of nitrogen (N_2_) fixing (*Azotobacter chroococum*), P-solubilizing (*Paenibacillus tylopili*) and K-solubilizing (*Bacillus decolorationis*) bacteria and an archaeal formulation based on *Halolamina pelagica* for drought alleviation developed at ICAR-NBAIM, Mau were evaluated along with the bacterial cultures. The detailed treatments are presented in Supplementary Table 1. The experiments were conducted in the farms of ICAR-NBAIM, Mau. The initial nutrient status of the field was as follows—organic carbon: 0.48%, N: 164.19 kg ha^−1^, P: 47.12 kg ha^−1^, K: 143.76 kg ha^−1^, Fe: 11.30 ppm; Zn: 1.32 ppm. All treatments received recommended dose of N (15 kg ha^−1^) and P (20 kg ha^−1^) in the form of urea and SSP. Chickpea var.PUSA362was used for the experiment. The experiment was conducted in 4.0 m × 3.0 m plots with randomized complete block design (RCBD) including three replicates. Spacing between two rows was kept at 30 cm. Drought was imposed by withholding irrigations and only one irrigation at pre-flowering stage was provided to the plants. Inoculum for seed treatment was prepared as described earlier for pot experiments. Bacterial suspension (~ 10^8^ CFU mL^−1^) containing 1.5% CMC was sprinkled at the rate of 40 mL per kg Chickpea seeds, mixed thoroughly, air dried under shade and left for 45 min before sowing. Data on plant height, root and shoot dry weight, number of nodules, nodule fresh and dry weight per plant were recorded at 45 days after sowing while yield parameters like number of pods per plant and seed yield were recorded during harvesting. N, P, K, Fe and Zn in Chickpea shoots collected during the harvesting stage were also determined as described in the previous section.

### Statistical analyses

All experimental data were analyzed statistically. Microsoft (MS) Excel was used to calculate the mean values and standard errors using the observations from at least three replications. Graphs were prepared using the MS Excel. Wherever applicable, the mean values were compared through Duncan’s multiple range test (DMRT) at P ≤ 0.05 using SPSSv.16.0.

### Supplementary Information

Supplementary Table 1.

## Data Availability

The datasets used and/or analysed during the current study available from the corresponding author on reasonable request.
