# Supplementary material for: Chasmophyte associated stress tolerant bacteria confer drought resilience to chickpea through efficient nutrient mining and modulation of stress response
Source: Sci Rep. 2024 May 28;14:12189. doi: 10.1038/s41598-024-58695-3 (PMC11133442; doi:10.1038/s41598-024-58695-3)
Supplement: Supplementary file 1 — Supplementary Table 1. [file 41598_2024_58695_MOESM1_ESM.doc]

Supplementary table 1:

| **Treatments** | **Details** |
| --- | --- |
| T1 | Un-inoculated control (receiving only one irrigation) |
| T2 | Inoculated with  *B. paralicheniformis* L38 and received one irrigation |
| T3 | Inoculated with  *Pseudomonas* sp. LN75 and received one irrigation |
| T4 | Inoculated with  *Enterobacter hormachei* subsp. *xiangfengensis* LJ89 and received one irrigation |
| T5 | Inoculated with  *B. paramycoides* L17and received one irrigation |
| T6 | Inoculated with  *Micrococcus luteus* LA9and received one irrigation |
| T7 | Inoculated with archaea *(Halolamina pelagica)* and received one irrigation |
| T8 | Inoculated with BioNPK and received one irrigation |
